# The association between right ventricular free wall strain and exercise capacity for health check-up subjects

**DOI:** 10.1371/journal.pone.0173307

**Published:** 2017-03-13

**Authors:** Wei-Ting Chang, Yen-Wen Liu, Ping-Yen Liu, Chih-Hsin Hsu, Wei-Chuan Tsai

**Affiliations:** 1 Division of Cardiology, Department of Internal Medicine, Chi-Mei Medical Center, Yungkang Dist, Tainan, Taiwan; 2 Division of Cardiology, National Cheng Kung University Hospital, Tainan, Taiwan; 3 Department of Biotechnology, Southern Taiwan University of Science and Technology, Tainan, Taiwan; 4 Department of Internal Medicine, Cheng Kung University Hospital, Tainan, Taiwan; Freeman Hospital, UNITED KINGDOM

## Abstract

**Background:**

Right ventricular (RV) function has been found to be a major factor of exercise capacity in patients with heart failure. However, the role of RV function in exercise capacity in healthy subjects has not been well studied. This study aims to validate the role of RV strain derived from speckle tracking echocardiography for exercise capacity for health check-up subjects.

**Methods:**

This study prospectively recruited subjects from a routine health examination. All of them were symptom free. RV function represented by RV strain was derived from speckle tracking echocardiography in addition to traditional echocardiography parameters. Functional capacity was determined by a symptom limited treadmill exercise test with the Bruce protocol.

**Results:**

Among 164 recruited subjects (age 52.2 ±9.2 years, 66.4% male), 32 subjects represented impaired functional capacity (MET<**8**), which was significantly correlated with age, left ventricular mass index, left ventricular filling pressure (E/e’), global longitudinal strain of the left ventricle (LVGLS) (-16.0±2.5% vs. -18.9±3.8%, p < 0.001) and RV free wall strain (RVLS_FW) (-17.0±4.9% vs. -21.9±3.2%, p <0.001). After multivariate logistic regression, RVS_FW was an independent predictor for impaired functional capacity (OR 1.62, CI 1.32–1.98; p <0.001).

**Conclusions:**

In conclusion, RV strain is independently associated with exercise capacity for health check-up subjects. RV function is an important factor for functional capacity.

## Introduction

Exercise capacity, as reflecting cardiac function, has been known as a powerful predictor of mortality among patients with various diseases [1]. Although the association between exercise capacity and traditional echocardiographic parameters remains indeterminate, emerging imaging modalities can help distinguish patients with preserved or impaired exercise capacity [2–3]. Among these modalities, speckle tracking echocardiography (STE) has been used to detect occult myocardial dysfunction that may result in reduced exercise capacity [4–5]. However, given that most of the previous studies focused on heart failure [3], congenital heart disease [6–7], chronic obstructive lung disease or athletes [8–9] the value of STE in detecting exercise capacity impairment in apparently healthy subjects remains unknown. In the real world, patients with physical disabilities cannot complete exercise testing and the alternative pharmacologic stress may lead to adverse effects like arrhythmia or hypotension. Therefore, investigating an imaging tool to evaluate the cardiac function as well as exercise capacity is helpful. Herein, we aim to identify the role of STE in detecting subtle myocardial dysfunction and its associated changes of exercise capacity in health check-up subjects.

## Methods

### Data source

From April 2012 to August 2013, 197 subjects undergoing routine health examinations were prospectively recruited. In Taiwan, according to the general awareness of health and the availability of medical access, regular health check-up is popular and all of enrolled subjects were free from symptoms. Among them, 33 subjects were excluded due to documented hypertension, diabetes, symptomatic heart failure, atrial fibrillation, significant valvular heart disease (above moderate severity), left ventricular ejection fraction less than 45%, and coronary artery disease, determined by the positive treadmill results. Clinical information on co-morbidities, medical history, and current cardiovascular medication was obtained by a careful review of each patient's medical record. Exercise capacity was determined by a symptom limited treadmill exercise test using the Bruce protocol [10]. The procedure was ceased according to the endurance of each individual and it could be a hallmark of exercise capacity and a representation of subclinical diseases. As has been reported, preserved exercise capacity was defined as an energy expenditure of more than 8 metabolic equivalent of task (MET) [11]. The study protocol conformed to the ethical guidelines of the 1975 Declaration of Helsinki, and was approved by the Human Research and Ethics Committee (IRB number: A-ER-104-018) in Chi-Mei Medical Center, Tainan, Taiwan. The written informed consent was obtained from each patient.

### Imaging acquisition

In accordance with the recommendations of the American Society of Echocardiography [12] all subjects received both standard and speckling tracking echocardiography (Vivid E9; GE Vingmed Ultrasound AS, Horten, Norway) at rest, at least one hour separated from Treadmill test. The chamber dimensions and left ventricular mass index (LVMI) were measured using the two-dimensionally guided M-mode method. The right ventricle fractional area change (RVFAC) was measured with the apical 4-chamber view while LV ejection fraction (LVEF) was measured by both 2- and 4- chamber Simpson method. Biventricular diastolic function associated parameters (including isovolumic relaxation time (IVRT), isovolumic contraction time (IVCT), deceleration time (DT), trans-mitral early filling velocity (E) to atrial velocity (A) ratio and mitral E to early diastolic mitral annular velocity (e’) ratio) were also measured. Peak systolic pulse Doppler tissue imaging was performed at the tricuspid annulus (S’). RV dimensions were defined right ventricular (RV) mid cavity dimension in parastenal long axis (RVD1), right ventricular outflow tract dimensions at the proximal or subvalvular level (RVD2) and at the distal or pulmonic valve (RVD3). In the apical four chamber view, the basal (RVD4) and mid cavity (RVD5) RV minor dimensions, the RV longitudinal dimension (RVD6) and tricuspid annular plane systolic excursion (TAPSE) were also measured. In addition, pulmonary artery systolic pressure (PAP) was obtained by the summation of the estimated trans-tricuspid valve pressure and the estimated right atrial pressure. The myocardial performance index (MPI), also called the Tei index, was calculated by (IVRT + IVCT) / ejection time. Left atrial volume index (LAVI) was calculated by 0.85 * (the area in the 4-chamber view × the area in the 2-chamber view) / the average of vertical axis in the 4-chamber and in the 2-chamber view [13]. Echocardiogram readers who analysed the data were blinded to the result of treadmill and subjects' clinical information.

### Speckle tracking echocardiography analysis for deformation

**Fig 1:**
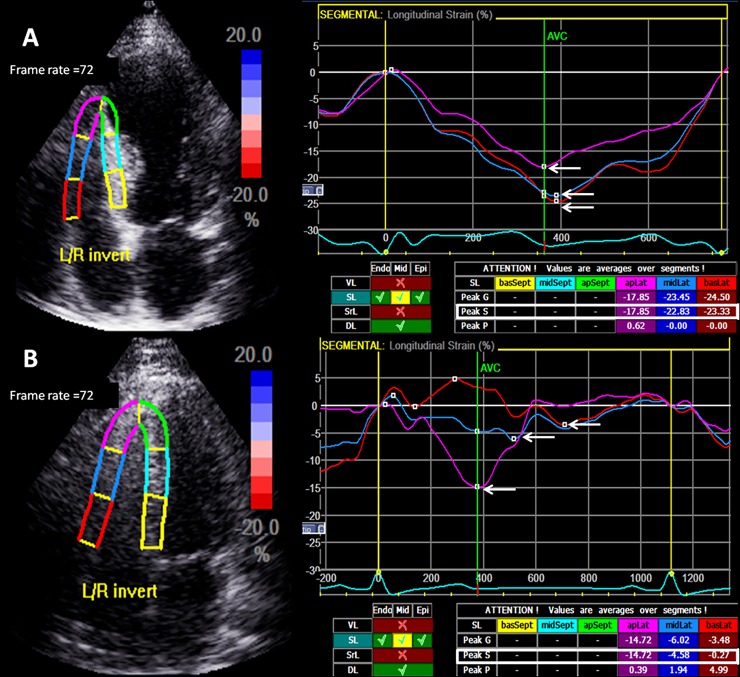
An example of measurement of right ventricular strain at free wall in a function preserved right ventricle (above) and the other function impaired right ventricle (below).

Standard apical 4-, 2- and 3-chamber views were recorded in the digital loops for deformation analysis of the LV, and an apical 4-chamber view focusing on the RV was used for RV deformation. The images were acquired with frame rates of 70–90 frame/s and stored for three cycles. The images were analysed off-line using computer software (EchoPAC 09, GE-Vingmed Ultrasound AS, Horten, Norway). As described previously [14], we used an automated function imaging software to measure the left ventricular peak systolic global longitudinal strain (LVGLS). In brief, the LVGLS was calculated automatically by the software after defining the timing of the aortic valve closure. RV deformation was measured using the two-dimensional STE in the apical 4-chamber view. Right ventricular free wall longitudinal strain (RVLS_FW) and strain rate were derived from the average of three regional strains comprising the lateral wall (Fig 1).

### Reproducibility

Using Bland-Altman limits of agreement and interclass correlation coefficients, 20 subjects were randomly selected to assess intra- and inter-observer variability.

### Statistical analysis

Differences among patients were compared using Student’s *t* tests for normally-distributed continuous variables, non-parametric test for non-normally distributed continuous variables and *x*^2^ tests for categorical variables. Factors with *p* <0.1 based on the univariate analyses were included in the multivariate logistic regression analyses. Multivariate models were developed with stepwise inclusion and exclusion at a significance level of 0.1. To separately discuss RVLS_FW as a continuous or a categorical variable with a cut-off value, model 1 and model 2 were applied respectively. In addition, correlations between variables were assessed with a univariate linear regression analysis. Receiver operating characteristic (ROC) curve analysis was used to determine the optimal cutoff values of RV strain in subjects with preserved or impaired functional capacity. The area under curves (AUC) of E/e’, age and RVLS_FW were also calculated. The best cutoff value was defined as the point with the highest sum of sensitivity and specificity. A *p* value of less than 0.05 was considered to be statistically significant. All analyses were performed with SPSS version 18 for Windows (SPSS Inc., Chicago, IL, USA).

## Results

### Clinical and echocardiographic characteristics of subjects with preserved and impaired exercise capacity

A total number of 164 subjects (age 52.2 ±9.2 years, 66.4% male) were enrolled. Among them, 32 (19.5%) presented impaired functional capacity (MET<8), which was significantly correlated with age, LVMI and left ventricular filling pressure (E/e’) while pulmonary artery systolic pressure was insignificantly increased. The other parameters of LVEF, RV dimensions, RVFAC and LAVI failed to discriminate the impaired functional capacity. Regarding to speckle tracking image, both global longitudinal strain of left ventricle (GLS) (-16.0±2.5% vs. -18.9±3.8%, p < 0.001) and RV free wall strain (RVS_FW) (-17.0±4.9% vs. -21.9±3.2%, p <0.001) were significantly lower in subjects with exercise intolerance (Tables 1 and 2).

**Table 1:** Comparison of clinical characteristics between subjects with preserved (MET ≥8) and impaired functional capacity (MET <8).

MET	≥8	<8	p-value
Patients No.(n)	132	32	
Age (y/o)	51.9±10.5	62.2±7.7	**0.001**
Male(n,%)	91 (68.9%)	18 (56.2)	0.55
BSA (kg/m2)	1.5±0.2	1.4±0.15	0.2
Exercise time (min)	9.1±1.4	4.2+1.1	**0.001**
SBP (mmHg)	136.7±18.8	134.7±29.4	0.25
DBP (mmHg)	83.6±12.8	83.9±13.5	0.9
Heart rate (beats/min)	81.9±13.3	82.4±14.2	0.87
HbA1c (%)	6.0±0.7	6.1±0.5	0.28

Data are expressed as mean ± SD. BSA = body surface area; SBP = systolic blood pressure; DBP = diastolic blood pressure.

**Table 2:** Comparison of clinical and echocardiographic parameters between subjects with preserved (MET ≥8) and impaired functional capacity (MET <8).

MET	≥8	<8	p-value
**Left heart dimension and function**			
LVMI (g/m2)	83.2±27.2	90.3±43.5	**0.04**
LVEF (simpson’s, %)	61.3±8.2	65.4±7.6	0.46
LVGLS (%)	-18.9±3.8	-16.0±2.5	**0.001**
IVRT (ms)	94.8±23.1	89.1±16.0	0.17
DT (ms)	192.9±65.4	183.2±48.8	0.14
E/A	1.0±1.4	1.1±0.8	0.22
E/e’(mean)	7.3±2.4	9.2±4.2	**0.02**
LAVI (ml/m2)	26.89±3.1	27.04±2.6	0.12
Right heart dimension*			
RVD1 (cm)	2.7±0.3	2.8±0.4	0.16
RVD2 (cm)	2.7±0.6	2.6±0.5	0.24
RVD3 (cm)	2.0±0.4	1.9±0.4	0.61
RVD4 (cm)	2.2±0.5	2.2±0.3	0.44
RVD5 (cm)	1.9±0.5	1.9±0.4	0.7
RVD6 (cm)	5.3±0.8	5.3±0.7	0.91
RV thickness (cm)	0.5±0.12	0.5±0.17	0.18
**Right heart function**			
PAP (mmHg)	17.5±9.8	20.9±10.73	0.06
FAC (%)	69.5±10.1	69.2±11.9	0.88
TAPSE (cm)	1.7±3.1	1.9±0.5	0.73
RV_MPI_(ms)	0.3±0.1	0.4±0.1	0.12
S’	13.1±2.4	12.6±2.8	0.32
RVLS_FW	-21.9±3.2	-17.0±4.9	**0.001**
RVLS_FW_basal_	-22.5±3.7	-15.7±4.2	**0.001**
RVLS_FW_middle_	-21.4±3	-16.8±8.9	**0.001**
RVLS_FW_apical_	-20.1±5.6	-18.8±8.8	**0.001**
RV E/A	0.7±0.3	0.9±1.0	0.12
RV_DT_ (ms)	160.6±45.7	155.8±38.4	0.45
RV_IVRT_ (ms)	55.8±23.2	57.4±33.1	0.14

Data are expressed as mean ± SD. LVMI = Left ventricular mass index; LVEF = left ventricular ejection fraction; LVGLS = LV global longitudinal strain (average of 17 segments); IVRT = isovolumic relaxation time; DT = deceleration time; E/A = trans-mitral valve E to A velocity ratio; E/e’ = mitral early filling velocity to early diastolic mitral annular velocity ratio; LAVI = left atrial volume index; PAP = pulmonary artery systolic pressure; FAC = right ventricle fraction area change; TAPSE = tricuspid annular plane systolic excursion; RV_MPI_ = RV myocardial performance index; S’ = peak systolic velocity of lateral tricuspid annulus in tissue Doppler; RVLS_FW = right ventricular longitudinal strain free wall (average of three segments); RVLS_FW_basal_ = right ventricular longitudinal strain free wall (basal segment)_;_ RVLS_FW_middle_ = right ventricular longitudinal strain free wall (middle segment)_;_ RV:S_FW_apical_ = right ventricular longitudinal strain free wall (apical segment); RV_IVRT_ = RV interventricular relaxation time.

*Right ventricular (RV) mid cavity dimension in parastenal long axis (RVD1). Right ventricular outflow tract (RVOT) dimensions at the proximal or subvalvular level (RVD2) and at the distal or pulmonic valve (RVD3), basal (RVD4) and mid cavity (RVD5) RV minor dimensions and the RV longitudinal dimension (RVD6).

### The independent factors for exercise capacity

**Fig 2:**
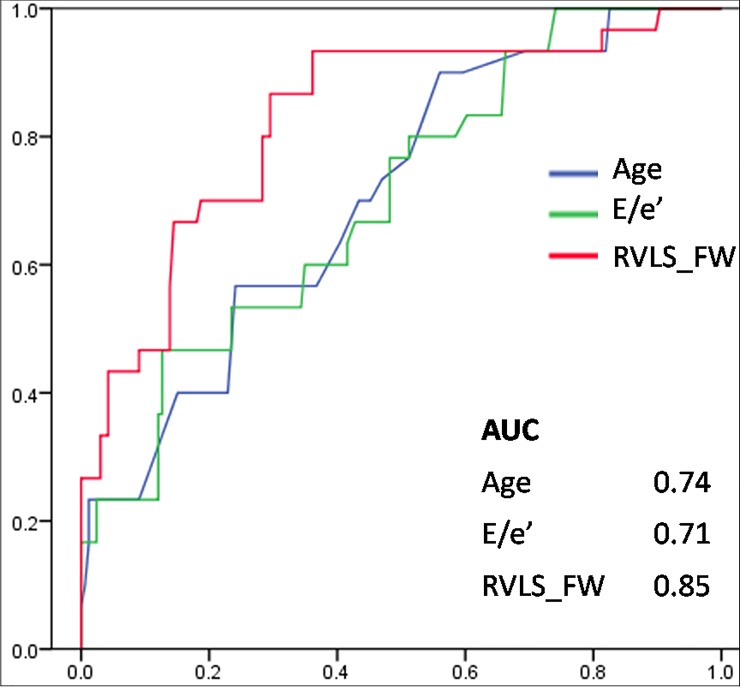
The areas under the ROC curves for the predictor of impaired functional capacity were 0.74 for age, 0.71 for E/e’ and 0.85 for RVLS_FW. Abbreviations as described in Tables 1 and 2.

In the logistic multivariate regression analysis with stepwise inclusion and exclusion, age (OR 1.27, CI 1.12–1.44; p = 0.011), E/e’ (OR 1.27, CI 1.04–1.54; p = 0.001) and RVLS_FW (OR 1.62, CI 1.32–1.98; p = 0.001) were significantly correlated with impaired functional capacity (Table 3). Among these, RVLS_FW presented the highest area under the ROC curve as 0.79 for a diagnosis of impaired functional capacity (Fig 2). According to the results of the ROC curve, we applied -18% of RVLS_FW as a cutoff point and the sensitivity and specificity were 73.4% and 74.9%, respectively. Using -18% of RVLS_FW as categorical variable instead of a continuous variable, RVLS_FW>-18% exhibited specific ability in discriminating subjects with impaired functional capacity in model 2 of the multivariate logistic regression (OR 18.92, CI 4.79–74.5; p = 0.001).

**Table 3:** Univariate and multivariate logistic regression of clinical and echocardiographic parameters in functional capacity with stepwise inclusion and exclusion.

	Univariate Analysis		Multivariate Analysis			
			Model 1		Model 1	
	HR (95% CI)	*P* Value	HR (95% CI)	*P* Value	HR (95% CI)	*P* Value
**Clinical parameters**						
Age	1.13	0.001	1.27	0.011	1.21	0.01
	(1.08–1.4)		(1.12–1.44)		(1.09–1.32)	
**Left heart parameters**						
LVGLS	1.29	0.001				
	(1.02–1.59)					
LVMI	1.02	0.001				
	(1.01–1.05)					
E/e’	1.31	0.001	1.27	0.001	1.29	0.001
	(1.06–1.4)		(1.04–1.54)		(1.11–1.51)	
**Right heart parameters**						
PAP	1.08	0.001				
	(1.03–1.13)					
RVS_FW	1.60	0.001	1.62	0.001		
	(1.31–1.96)		(1.32–1.98)			
RVS_FW>-18%	21.76	0.001			18.92	0.001
	(6.33–74.81)				(4.79–74.5)	

Abbreviations as described in Tables 1 and 2.

### The diagnostic impact of RVLS_FW in the significant population

**Fig 3:**
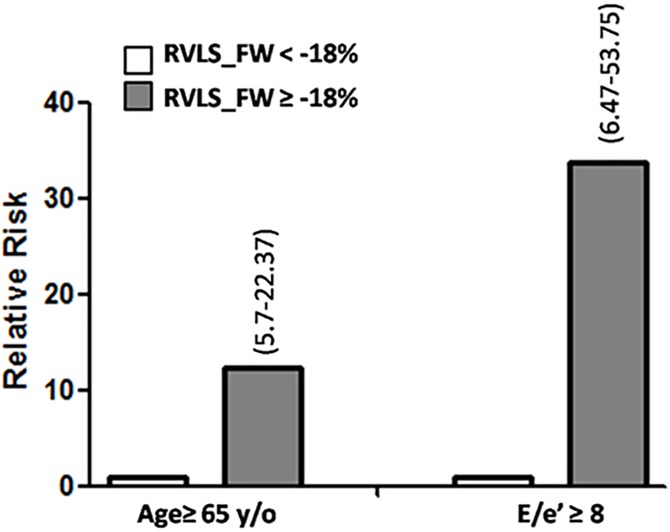
Relative risks of impaired functional capacity in subjects with old age (≧ 65 y/o) and upper limit of estimated wedge pressure (E/e’≧8) who have relatively preserved (< -18%) or declined RVLS_FW (≧ -18%). Abbreviations as described in Tables 1 and 2.

We also noticed negative correlations between MET to age, E/e’ and RVLS_FW in the linear regression (S1 Fig). To study the diagnostic impact of RVLS_FW in these specific populations, we focused on subjects with old age (age ≥ 65 y/o) and upper limit of estimated wedge pressure (E/e’ ≥ 8). Notably, the odds ratio increased dramatically to 12.37 (CI: 5.7–22.37, p = 0.001) and 33.75 (CI: 6.47–53.75, p = 0.001), respectively (Fig 3).

### The regional differences in LV and RV in subjects with impaired exercise capacity

For the assessment of the territorial correlation between regional strain and functional capacity, we separately discuss the RV free wall in basal, middle, and apical segments. Compared with subjects with preserved function capacity, the subjects with impaired functional capacity were associated with lower strain all over the apical, middle and basal segments (Table 2). However, in the multivariate analysis only the basal segment was significantly associated with impaired functional capacity (OR 1.14, CI 1.04–1.23; p = 0.004) (S1 Table). For the 17 segments of regional strain of the left ventricle, the basal segments were related to a significantly lower strain compared with the apical segments (S2 Table). After the multivariate analysis, only the basal anterolateral (OR 1.21, CI 1.04–1.41; p = 0.01) and inferolateral walls (OR 1.24, CI 1.07–1.43; p = 0.003) were highly correlated to the functional capacity (S3 Table).

### Reproducibility of LV and RV strain

The echocardiographic images of 20 randomly selected subjects were analysed by two readers for a total of three times each. Each measurement was taken at fifteen-minute intervals. Readers could select the best cardiac cycle by themselves and were blinded to previous measurements. The intra- and inter-observer interclass correlation coefficients for RVLS_FW were 0.95 (0.78–0.96) and 0.94 (0.91–0.97), respectively. For LVGLS, the intra- and inter-observer interclass correlation coefficients were 0.87 (0.66–0.92) and 0.92 (0.84–0.97). The mean intra- and inter-observer differences for RVLS_FW were -0.84 ± 0.31 (-1.91 to 3.88% limit of agreement) and -0.85 ± 0.42 (-2.63 to 4.3% limit of agreement), respectively. For LVGLS, the mean intra- and inter-observer differences were -0.78 ± 0.31 (-2.65 to 2.75% limit of agreement) and -0.78 ± 0.32 (-1.94 to 3.13% limit of agreement).

## Discussion

There are three main findings of the present study: (1) impaired RV strain was significantly correlated to impaired functional capacity; (2) the cut-off value of -18% RV strain represented the best discriminative value; and finally, (3) among regional strains, the impairment of the basal RV and basal LV strain were most significantly associated with the functional decline.

Exercise capacity is known to be an important prognostic factor in not only patients with cardiovascular disease but also among healthy persons [1,3]. In a prospective study enrolling 6213 consecutive men referred for treadmill exercise testing, MET was the strongest predictor of the risk of death among both normal subjects and those with cardiovascular disease [1]. A cut-off value of 8 MET was regarded reliable to differentiate patients with preserved or impaired METs. A correlation between cardiac dysfunction and functional capacity can help research of both physiology and the potential clinical applications. However, traditional echocardiographic measurement like ejection fraction failed to discriminate the subtle changes of cardiac function [15]; STE, though, can detect very early myocardial impairment [4]. In patients with heart failure, LVGLS has been reported as correlating to exercise capacity in failing hearts with preserved and reduced ejection fraction [3]. In these cases, LVGLS was superior to other myocardial function parameters, including RV strain and E/e’, to identify patients with decreased peak maximal oxygen uptake.

Compared with LV, RV adapts to exercise and has been regarded as a determinant for symptom severity and survival in patients with congenital heart disease as well as pulmonary hypertension [16,17]. Beyond the additional value in left heart failure and valvular heart disease, RV plays a pivotal role in the regulation and compliance of the pulmonary vascular system [18,19] With exercise, RV dysfunction results in the increase of vascular pressures and impairment of oxygen exchange. However, studies focusing on RV function and functional capacity in apparently healthy subjects remain lacking. In a recent study, Chia and colleagues compared the RV functions in rest and post-exercise [20]. Using STE, they indicated that with aging, both systolic and diastolic RV augmentation decreased. However, most of the LV parameters were missing and the relationship between impaired functional capacity and RV dysfunction could not be determined. In contrast, our study focused on identifying a sensitive and specific parameter to detect the subtle myocardial dysfunction, which reflects the exercise capacity. Our study thus found that RV function is an independent predictor for exercise capacity. Though LVGLS was also significantly correlated with functional capacity, we deem the discriminative value of RVLS_FW to be superior due to the course of disease progression. Being supplied mainly by one coronary artery, RV is more vulnerable to ischemia than LV [21]. Playing a pivotal role in the pulmonary circulation, inflow to the heart and fluid reservoir, RV is thus closely associated with exercise capacity

With the decline of MET, we found positive correlations with increased left atrial pressure (E/e’), age and the severity of impaired RVLS_FW. These factors may be considered as the earliest predictors of decreasing functional capacity. According to the close correlation of wedge pressure and right ventricular pressure to the preload and afterload of the pulmonary circulation system [22], we focused on the impact of RV strain in patients with upper limit of estimated left atrial pressure (E/e’). Interestingly, in these groups, the discriminative power of RVLS_FW increased significantly. This finding indicates the specific value of RV strain in the applications of certain relatively risky populations.

Previous studies have suggested various normal ranges of RV strain, including -21% for primary outcome prediction in patients with LV systolic heart failure [23] and -18% in patients with arrhythmogenic RV cardiopathy [24]; however, none of those can claimed to be accurate owing to limited evidences. In our study, we used a cut-off point of ≤-18% according to the median value of RV strains. After dividing the population by -18% RV strain, the patients with preserved RV strain (≤-18%) had significantly better exercise capacity. Notably, in subjects with MET>8, the averaged RVLS_FW was measured as -21.9±3.2% which was relatively lower compared with the normal RV strain in previous literature [21]. Nevertheless, though subjects with documented hypertension, diabetes, symptomatic heart failure, atrial fibrillation, significant valvular heart disease, decreased LVEF and coronary artery disease were excluded in our study, we were not attempting to enroll subjects defined as completely normal but with relatively preserved exercise capacity. This may explain why our values of RVLS_FW differed from the previously reported ones.

Despite the RVLS_FW decline in all three segments in patients with impaired functional capacity, we found that only the basal segment was significantly associated with functional decline. The structure of RV is complex [20], with the apex being heavily trabeculated and relatively immobile. In previous studies, RV strain measured by tissue Doppler and STE both decreased with age, especially in the basal segments [21,25]; and patients with Brugada syndrome and arrhythmogenic RV cardiomyopathy were observed to have basal and mid segments more involved than the apex [26]. Conversely, in patients with post atrial septal defect repair, lower apical strain values correlated with functional capacity [7]. Also, in athletes after isometric stress, the two ventricles showed particular myocardial deformation around the apex [27]. However, in these two specific populations, the significant changes over apical segments may be ascribed to volume overload. Regarding LV, our results indicate that the basal segments presented a significant lowering of strain compared with the apical segments. After the multivariate analysis, only the basal anterolateral and inferolateral walls were highly correlated to the functional capacity. Correspondingly, using the Duke Activity Status Index, LVGLS (especially the inferolateral segments) had a strong linear association with estimates of functional capacity in patients with and without preserved ejection fraction [28]. Because many factors can affect segmental strains [29], the roles of both LV and RV segmental strains in determination of exercise capacity require further investigation. We recognize several limitations with this study. First, only a small number of subjects presented impaired functional capacity. Even though RVLS_FW was significantly correlated to functional decline, the statistical findings may be over-fitted. Secondly, RVLS_FW was observed to decrease with increasing age but in multivariate logistic regression, RVLS_FW was still superior in the correlation to the functional capacity. Third, according to the exclusion criteria, patients with mildly impaired LVEF between 45–50% were also enrolled but only accounted a very small amount. Fourth, Using Treadmill to monitor exercise capacity may not as optimal as using maximum O2 uptake, which is regarded as the best measure for exercise capacity currently. Fifth, in our study, most of the enrolled subjects were at their middle ages while our results may not be applied in the younger subjects. Also, using the cut-off value of 8 METs to differentiate exercise capacity may not fit in women who generally have lower physical tolerances [30]. Finally, there is no definitive normative range of RV function for STE evaluation while more studies are required.

## Conclusions

RV strain is independently associated with functional capacity in health check-up subjects. RV function should be regarded as an important factor for exercise capacity.

## Supporting information

S1 Fig **(A)** The linear regression of age and metabolic equivalent of task (MET) **(B)** The linear regression of E/E’ and MET **(C)** The linear regression of RVLS_FW and MET.
(TIF)

S1 Table Univariate and multivariate logistic regression of regional right ventricular free wall strain. (DOCX)

S2 Table Comparison of regional left ventricular strain between patients with preserved (MET ≥8) and impaired functional capacity (MET <8). (DOCX)

S3 Table Univariate and multivariate logistic regression of regional left ventricular longitudinal strain (LVLS). (DOCX)
