# Supplementary figures and images for: The association between right ventricular free wall strain and exercise capacity for health check-up subjects
Source: PLoS One. 2017 Mar 13;12(3):e0173307. doi: 10.1371/journal.pone.0173307 (PMC5348016; doi:10.1371/journal.pone.0173307)

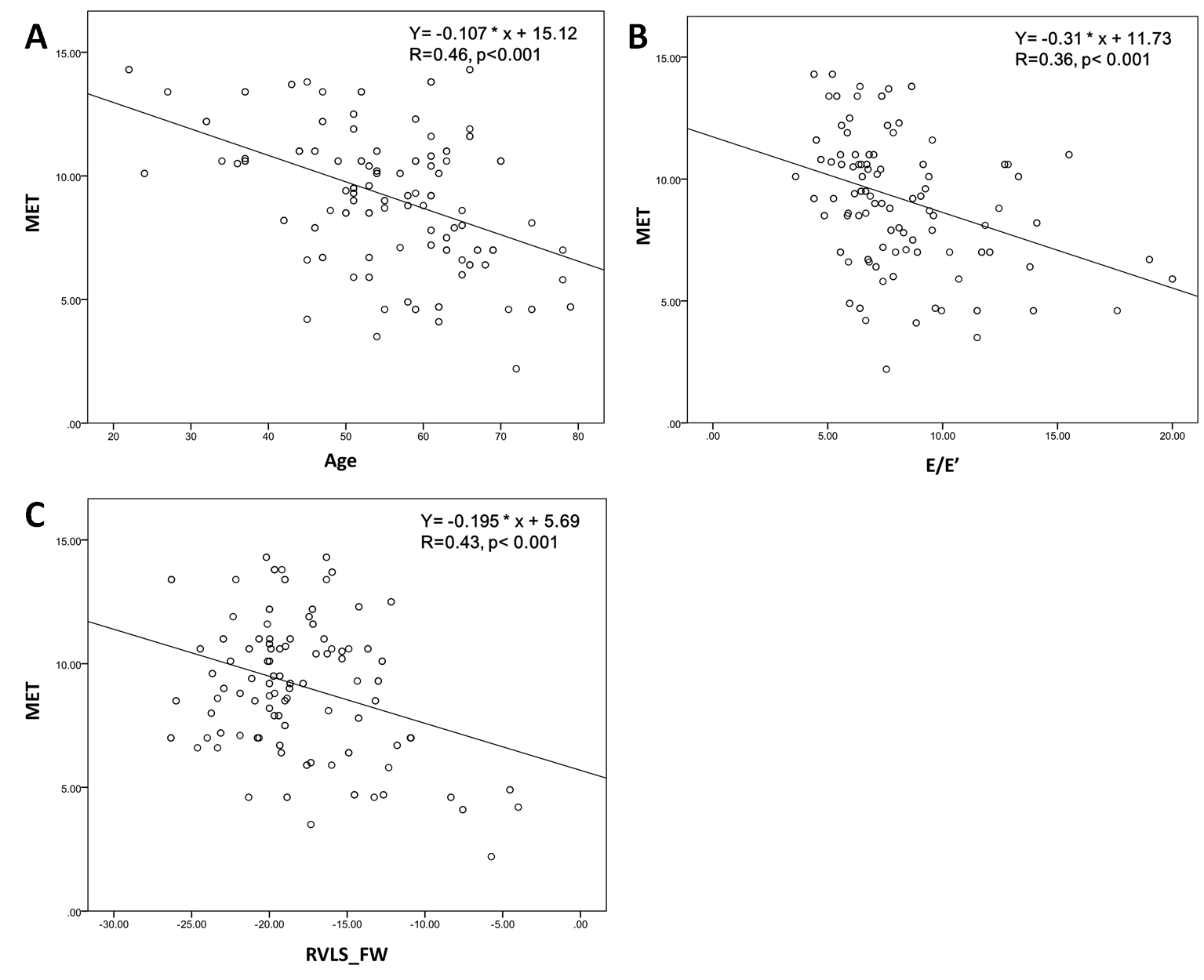

Supplement: S1 Fig — (A) The linear regression of age and metabolic equivalent of task (MET) (B) The linear regression of E/E’ and MET (C) The linear regression of RVLS_FW and MET. (TIF) [file pone.0173307.s001.tif]
