# Supplementary material for: The association between right ventricular free wall strain and exercise capacity for health check-up subjects
Source: PLoS One. 2017 Mar 13;12(3):e0173307. doi: 10.1371/journal.pone.0173307 (PMC5348016; doi:10.1371/journal.pone.0173307)
Supplement: S1 Table — (DOCX) [file pone.0173307.s002.docx]

**Supplement Table 1. Univariate and multivariate logistic regression of regional right ventricular free wall strain**

|  | Univariate Analysis | | **Multivariate Analysis** | |
| --- | --- | --- | --- | --- |
|  | HR (95% CI) | *P* Value | HR (95% CI) | *P* Value |
| RVS_FW_basal_ | 1.24(1.14-1.35) | 0.001 | 1.14(1.04-1.23) | 0.004 |
| RVS_FW_middle_ | 1.4(1.23-1.6) | 0.001 | 1.31(0.96-1.77) | 0.088 |
| RVS_FW_apical_ | 1.25(1.14-1.38) | 0.001 | 1.02(0.8-1.28) | 0.887 |

Abbreviations as described in Table 2.
