# Supplementary material for: The association between right ventricular free wall strain and exercise capacity for health check-up subjects
Source: PLoS One. 2017 Mar 13;12(3):e0173307. doi: 10.1371/journal.pone.0173307 (PMC5348016; doi:10.1371/journal.pone.0173307)
Supplement: S2 Table — (DOCX) [file pone.0173307.s003.docx]

**Supplement Table 2. Comparison of regional left ventricular strain between patients with preserved (MET ≥8) and impaired functional capacity (MET <8)**

| **MET** | **≥8** | **<8** | **p-value** |
| --- | --- | --- | --- |
| LVLS basal anterior | -16.4±4.3 | -12. 9±6.3 | 0.001 |
| LVLS basal anteroseptal | -16.1±4.5 | -8.4±8.7 | 0.001 |
| LVLS basal inferoseptal | -18.1±3.7 | -16±4.3 | 0.004 |
| LVLS basal inferior | -13.2±10.2 | -10.1±8.9 | 0.001 |
| LVLS basal inferolateral | -17.7±5.2 | -14.3±5.5 | 0.001 |
| LVLS basal anterolateral | -17.7±5.1 | -12.9±7.6 | 0.001 |
| LVLS mid anterior | -20.4±6.4 | -17.4±8.1 | 0.01 |
| LVLS mid anteroseptal | -19.9±3.1 | -14.8±9.2 | 0.001 |
| LVLS mid inferoseptal | -20.7±3.3 | -19.4±5.3 | 0.07 |
| LVLS mid inferior | -16.7±7.6 | -12.5±10.5 | 0.092 |
| LVLS mid inferolateral | -19±5.3 | -15.3±7.7 | 0.001 |
| LVLS mid anterolateral | -19.9±5.1 | -14.7±6.6 | 0.008 |
| LVLS apial anterior | -21.1±10.2 | -18.3±13.7 | 0.16 |
| LVLS apial anteroseptal | -21.1±7.9 | -20.1±5.4 | 0.51 |
| LVLS apial inferoseptal | -19.6±6.6 | -17.7±9 | 0.15 |
| LVLS apial inferior | -20.8±7.3 | -18.3±5.2 | 0.06 |
| LVLS apex | -21.9±3.9 | -19.1±6.7 | 0.17 |

LVLS= left ventricular longitudinal strain
