# Supplementary material for: The association between right ventricular free wall strain and exercise capacity for health check-up subjects
Source: PLoS One. 2017 Mar 13;12(3):e0173307. doi: 10.1371/journal.pone.0173307 (PMC5348016; doi:10.1371/journal.pone.0173307)
Supplement: S3 Table — (DOCX) [file pone.0173307.s004.docx]

**Supplement Table 3. Univariate and multivariate logistic regression of regional left ventricular longitudinal strain (LVLS)**

|  | Univariate Analysis | | **Multivariate Analysis** | |
| --- | --- | --- | --- | --- |
|  | HR (95% CI) | *P* Value | HR (95% CI) | *P* Value |
| LVLS basal anterior | 1.15(1.06-1.26) | 0.001 | 1.07(0.9-1.25) | 0.43 |
| LVLS basal anteroseptal | 1.12(1.05-1.19) | 0.001 | 0.97(0.79-1.2) | 0.81 |
| LVLS basal inferoseptal | 1.15(1.04-1.26) | 0.006 | 1.05(0.83-1.33) | 0.65 |
| LVLS basal inferior | 1.03(0.99-1.06) | 0.09 |  |  |
| LVLS basal inferolateral | 1.22(1.12-1.33) | 0.001 | 1.24(1.07-1.43) | 0.003 |
| LVLS basal anterolateral | 1.17(1.09-1.26) | 0.001 | 1.21(1.04-1.41) | 0.01 |
| LVLS mid anterior | 1.05(1-1.11) | 0.03 | 0.97(0.75-1.25) | 0.81 |
| LVLS mid anteroseptal | 1.22(1.09-1.36) | 0.001 | 1.11(0.84-1.47) | 0.46 |
| LVLS mid inferolateral | 1.09(1.03-1.16) | 0.002 | 0.97(0.75-1.25) | 0.82 |
| LVLS mid anterolateral | 1.14(1.06-1.21) | 0.001 | 1.05(0.92-1.21) | 0.47 |

LVLS= left ventricular longitudinal strain
